# Thrombotic microangiopathic anemia in patients with sickle cell disease and its variants during a vaso-occlusive crisis

**DOI:** 10.1016/j.rpth.2025.102734

**Published:** 2025-03-18

**Authors:** Wouhabe Bancheno, Donna Alexander, Mekdem Melaku, Bary Malik

**Affiliations:** 1 Division of General Internal Medicine, Department of Internal Medicine, Faculty of Medicine, Howard University Hospital, Washington, DC, USA; 2 Louis Stokes Health Sciences Library, Washington, DC, USA

## 1 Introduction

Activation of thrombotic and complement pathways is increased in patients with sickle cell disease (SCD) experiencing vaso-occlusive crisis (VOC). Activated endothelial cells release high levels of von Willebrand factor (VWF), with concomitant accumulation of ultralarge VWF multimers, correlating with hemolysis and free hemoglobin burden in those patients. In some patients, the ultralarge VWF tethering to active endothelial surfaces in small vessels may facilitate initiation of thrombotic microangiopathic anemia (TMA). TMA in SCD is a microvascular disorder characterized by thrombocytopenia and microangiopathic hemolytic anemia [1]. Acquired thrombotic thrombocytopenic purpura (aTTP) with impaired or severe ADAMTS13 (a disintegrin and metalloprotease with thrombospondin type 1 motif, member 13) deficiency (<10%), a form of TMA, can occur at the time of VOC in patients with SCD. Some researchers suggest that TMA occurring during VOC, irrespective of ADAMTS13 level, presents a distinct diagnosis similar to classical aTTP, while others view the condition as part of the spectrum of severe SCD [2]. In this systematic review, we aimed to evaluate the occurrence, clinical presentation, diagnosis, and treatment strategies of TMA during VOC in SCD.

## 2 Study Methods

We aimed to evaluate the literature about the occurrence of TMA including aTTP in patients with SCD and its variants during VOC, the presentation, diagnosis, treatment, and its outcomes. We searched PubMed, Clinical Key, Ovid, and Google Scholar using terms related to TMA, aTTP, and SCD. We included case reports and series of suspected or confirmed TMA including aTTP in patients over 16 years old, excluding pregnant women. We also incorporated scientific letters and abstracts, along with a patient treated at our facility. We defined TMA during VOC on the basis of significant thrombocytopenia, microangiopathic hemolytic anemia, and superelevated lactate dehydrogenase (LDH) level in the absence of alternative diagnosis. We defined aTTP as TMA with ADAMTS13 activity <10%. Two authors screened articles for relevance, and data from included studies were tabulated covering publication details, patient demographics, clinical presentation, time of diagnosis, laboratory findings, treatments, hospitalization duration, and patient outcomes. This study had no intervention, hence no ethical clearance was required. Informed consent was obtained for the patient data retrieved from our facility.

## 3 Results

We retrieved 16 full-length articles, 3 abstracts, and 1 case from our institution, covering the period 1988 to 2023 [[3], [4], [5], [6], [7], [8], [9], [10], [11], [12], [13], [14], [15], [16], [17], [18], [19], [20], [21]]. Tables 1 and 2 summarize the findings of 29 cases analyzed. The male-to-female ratio was 2:1 and cases were mainly from the United States, followed by Bahrain. The age of the 19 individually reported cases ranged from 16 to 48 years, with a mean of 30.5 years (SD ±0.2 years). In the series by Shome et al. [3], age range was 18 to 51 years, with a mean of 30.8 years. Of 29 patients, 20 (69%) had sikcle cell hemoglobin (HgbSS), 3 with hemoglobinS and C (HgbSC), 2 with sickle cell trait, 3 with hemoglobin S and β^+^ (Hgb Sβ^+^) and 1 with sickle-unspecified thalassemia.

**Table 1:** List of patients with thrombotic microangiopathic anemia and sickle cell disease and its variants who had ADMT13 test.

Reference, country, year	Clinical presentations	Main diagnostic workup results	Treatment and outcome
Shelat [4], USA, 2010	16 yo, SS F, headache, body pain, hypoxia, purpura, ecchymosis fever of 38.3 °C	Hgb nadir 7 g/dL, platelet nadir 70, 000/mm^3^; schistocytes present; LDH peak >6000 U/L, no DIC or sepsis; ADAMTS13 activity normal.	Received RCE for ACS, PLEX, antibiotics; alive and laboratory values normalized on discharge
Aiken et al. [5], USA, 2022	27 yo F, Hgb SS, pain crisis; hypoxia saturation of 70% on next day	Hgb nadir 6.3 g/dL, platelet nadir 91,000/mm^3^, schistocytes present; LDH peak 2646 U/L; ADAMTS13 64.9% (normal low >66.8%).	ICU admission; got PRBC, antibiotics, high-dose intravenous steroid after PLEX; improved, alive
Kodali et al. [6], USA, 2019	45 yo F, Hgb SC, severe pain crisis, day 3, confused, tachycardic	Hgb nadir 7.1 g/dL, platelet nadir 49,000/mm^3^ schistocytes present; LDH peak 4300 U/L; Cr 1.89 mg/dL, ADAMTS13 99%; day 5 MRI brain CVA.	PRBC 1 unit; RCE for CVA, PLEX for TTP; discharged alive; laboratory values improved except high LDH
Aggarwal et al. [7], Canada, 2021	20 yo M sickle cell trait, fatigue and confusion, abdominal pain, melena, and emesis; subsequent deterioration of mentation	Hgb 4.8 g/dL, platelet nadir 9,000/ mm^3^; schistocytes positive, LDH peak 3721 U/L, DAT negative; Cr 2.94 mg/dL, ADAMTS13 <10%, inhibitor antibody positive.	ICU admission needed; received high-dose steroid, PLEX, caplacizumab for recurrence; alive
Goldenberg et al. [8], USA, 2022	35 yo F SS; pain crisis, worsening change in mentation, fever, hypoxia; acute renal and liver failure	Hgb 5.2 g/dL, platelet nadir 52,000/mm^3^, schistocytes positive, LDH high, no DIC PLASMIC score 4; ADAMTS13 5%; CT head extensive dural venous sinus thrombosis.	ICU admission, vent, transfused with PRBC, received PLEX, heparin; laboratory and mental status change resolved; alive
Al-Juhani and Ayyub [9], Saudi Arabia, 2013	30 yo M SS; had fever, pain, headache, hypoxia, confusion	Hgb 6.5 g/dL; platelet nadir 36,000/mm^3^; schistocytes present; LDH peak 6996 U/L, ADAMTS13 62%, X**-**ray lower zone infiltrates.	ICU admission and vent, received RCE, PLEX, and antibiotics; laboratory values and patient improved; alive
Gangemi and Pickens [10], USA, 2015	48 yo F HgbS/β+–thalassemia, acute pain, fatigue; laboratory values and patient mentation deteriorated	Hgb 10.2 g/dL, platelet nadir 26,000/mm^3^; schistocytes present; LDH peak 3556 U/L; ADAMTS13 mild decrease 61% (>66% normal).	Platelet given for IVCF; received PRBC, PLEX, rituximab, and antibiotics; died of PCJ 3 mo later
Chonat et al. [11], USA, 2016	19 yo F SS; pain, fever, ACS; hemolysis, renal failure in next 24 h, seizure and mentation change on day 4	Hgb 3 g/dL, platelet nadir 3,100/mm^3^; schistocytes present; LDH peak >4000 IU/L, Cr 6.2 mg/dL; ADAMTS13 80%; sC5b-9 high; complement regulatory gene mutation.	Received RCE for ACS, PLEX for TMA day 4, eculizumab on day 11; laboratory values improved, discharged alive, atypical HUS per authors
This study, USA, 2023	29 yo M, SS thalassemia trait; generalized body pain, fever	Platelet nadir 52,000/mm^3^, Hgb nadir 5.7 g/dL; LDH peak 2304U/L, ADAMTS13 60%.	PLEX given on day 8, given PRBC, improved, alive

ACS, acute chest syndrome; AKI, acute kidney injury; ASA, aspirin; Cr, creatinine; CVA, cerebrovascular accident; CXR, chest x-ray; DAT, direct antiglobulin test; DIC, disseminated intravascular coagulation; DIC, disseminated intravascular coagulation; F, female; Hgb, hemoglobin; HIT, heparin induced thrombocytopenia; HUS, hemolytic uremic syndrome; ICU, intensive care unit; IVCF, inferior vena cava filter; LDH, lactate dehydrogenase; M, male; MOF, multiple organ failure; MRI, magnetic resonance imaging; PRBC, packed red blood cell; PLEX, plasma exchange; RCE, red blood cell exchange; RV, right ventricle; SC, sickle cell and C hemoglobin; SS, sickle cell hemoglobin;Sβ+, sickle cell and beta thalassemia hemoglobin; TMA, thrombotic microangiopathic anemia; TTP, thrombotic thrombocytopenic purpura; yo, years old.

**Table 2:** List of patients with thrombotic microangiopathic anemia and sickle cell disease and its variants who had no ADMT13 test.

Reference, country, year	Clinical presentation	Main diagnostic work up results	Treatment and outcome
Alsaghir et al. [12], Saudi Arabia, 2023	23 yo F SS; pain; respiratory failure, encephalopathy; rash, hypotension	Hgb nadir 5.5 g/dL; platelet nadir 27,000 mm^3^; schistocytes present, LDH peak 2000 U/L; Cr 2 mg/dL; lobar embolus; possible RV thrombus	Received RCE for FES/aTTP, did not respond; when given PLEX, responded; imaging and laboratory values resolved; alive
Chatiwala et al. [13], USA, 2006	48 yo M SS; pain, altered; worsening anemia	Hgb nadir 4.6 g/dL; platelet nadir 9000/mm^3^ schistocytes present; LDH peak 8772 U/L, AKI	Received PLEX from outset; improved mild renal insufficiency
Bolaños-Meade et al., [21], USA, 1999	44 yo SS M; vomiting, weak abdomen tender, fever, coma	Hgb nadir 5.5 g/dL; platelet nadir 21,000/mm^3^, schistocytes yes day 4, LDH 8033 U/L, Cr 2.3 mg/dL	Received PRBC, platelet, PLEX, antibiotics; discharged alive, but died of recurrence
Chehal and Taher [22], Lebanon, 2002	40 yo M sickle cell thalassemia; pain, fever, altered, developed MOF	Platelet nadir 38,000/mm^3^, schistocytes yes, LDH peak 7689 U/L; Cr 5.7 mg/dL, echo EF 35%	Vented in ICU; received RCE, condition improved; but deteriorated again, later PLEX improved, and discharged well
Chinowsky [14], USA, 1994	22 yo F, Hgb SC, pain, coma, and fever (on oral contraception)	Hgb nadir 6 g/dL; platelet nadir 53,000/mm^3^; schistocytes present, LDH peak 3147 U/L	Received RCE, steroid, PLEX, antibiotics; clinical resolution after PLEX
Lee et al. [15], USA, 2003	24 yo M, Hgb SC, pain, hypoxia, seizure, febrile	Hgb nadir 6.6 g/dL; platelet nadir 19,000/mm^3^, schistocytes yes; LDH peak 11,250 U/L	Received PRBC, antibiotics; later PLEX and dialysis; discharged alive
Prichard et al. [16], USA, 1988	37 yo M Hgb AS, epigastric pain, fever, stupor after FFP	Hgb nadir 8.21 g/dL, platelet 111,000/mm^3^, schistocytes yes, Cr 1.4 mg/dL, DIC negative	Given PRBC, steroid, ASA, dipyridamole, FFP; laboratory values and patient improved; alive
Geigel and Francis [17], USA, 1997	23 yo M SS; pain, vomiting, gum bleed, lethargy	Hgb 5.6 g/dL, platelet nadir 75,000/mm^3^, LDH high, schistocytes yes, Cr 3.1 mg/dL, coagulopathy	In ICU, received FFP, antibiotics, PRBC, and RCE; PLEX on day 4 with resolution
Vlachaki et al. [18], Greece, 2014	30 yo M HbSβ+, gum bleed, epistaxis, bone pain, agitated	Hgb 7.1 g/dL, platelet 7000/mm^3^, schistocytes yes on day 11, LDH peak 2000 U/l, DAT negative, DIC negative, day 11 CT showed brain hemorrhage	Received PRBC; steroid, IVIG, vincristine, and PLEX; after PLEX, hypertension, hematuria, headache, and CVA occurred and died
Shome et al. [3], Bahrain, 2013 (case series)	10 cases (9 HgbSS, 1 HgbSβ; mean age, 30.8 y, 8 M/2 F; referred for PLEX for MAHA; had chest pain, respiratory distress, fever; neurologic dysfunction in 9 (2 had CVA); oliguria in 4; MOF in 5 patients	Leukocytosis in 8 cases; Hgb mean nadir 7.3 g/dL; admission thrombocytopenia in 6; in 4, developed nadir rapidly after admission: platelet mean 36,500/mm^3^; schistocytes yes in all cases. LDH in all >1000 U/L; DAT negative in all cases; Cr high in 3; no DIC; culture negative except in 1 for *Escherichia coli* in blood, unknown source (without diarrhea and renal failure); CXR extensive infiltrates in all cases (ACS)	PRBC, before PLEX; RCE in all cases; used antibiotics; lack of response to transfusion prompted PLEX use; 9 patients recovered fully after PLEX. In 1, incomplete response to PLEX from new complications. On long follow-up, 1 recurrent episode and 1 death from another crisis without MAHA
Hinestroza and Can [19], USA, 2020	19 yo M SS, first time pain, fever, SOB tachycardia, and confused	Hgb nadir 6.8 g/dL, platelet initial normal, nadir 45,000/mm^3^ in 48 h, schistocytes difficult to tell; LDH peak unknown; HIT negative, CXR negative	Received PRBC transfusion, RCE not clear, PLEX no; alive after treated as atypical VOC vs TTP

aTTP, acquired thrombotic thrombocytopenic purpura; Cr, creatinine; CT, computed tomography; CVA, cerebrovascular accident; FES, fat embolism syndrome; Hgb, hemoglobin; ICU, intensive care unit; IVIG, intravenous immunoglobulin; LDH, lactate dehydrogenase; MAHA, microangiopathic hemolytic anemia; PLEX, plasma exchange; PRBC, packed red blood cell; RCE, red blood cell exchange; TMA, thrombotic microangiopathic anemia; TTP, thrombotic thrombocytopenic purpura; yo, years old.

Of the 29 cases, in 25 (86.2%), the admission reason was a pain crisis. One patient with sickle cell trait presented with left-sided face and hand numbness and was admitted as sickle cell crisis [16]. The remaining 3 had varied presentations: 1 with gastrointestinal symptoms [7]; another with vomiting, weakness, dark urine, and suspected urinary tract infection [21]; the third patient with postdental extraction gingival bleeding [18]. Twenty of the 29 cases (69%) had fever, while 27 (93.1%) showed neurologic symptoms including encephalopathy, coma, and seizures; of these, 4 experienced cerebrovascular accident, including 1 intracerebral hemorrhage [3,6,18]. A case showed brain ischemia and edema from suspected fat embolism syndrome (FES) [12], and dural venous sinus thrombosis occurred in 1 patient [8].

Of the 27 patients with Hgb results, the range was 4.6 to 10.2 g/dL and the mean nadir was 6.7 g/dL (SD ±1.2 g/dL). Schistocytes were found in 27 (93%) cases during nadir thrombocytopenia. In 2 cases, including our case, test results were not available [14]. Thrombocytopenia occurred in all patients, with a mean nadir platelet count of 43,000/mm^3^ (SD ±24,900/mm^3^) and range of 7000 to 111,000/mm^3^. Among 25 patients with LDH values, the mean was 4366.2 U/L (SD ±2470 U/L), with a range of 1229 to 11,250 U/L.

ADAMTS13 activity was assessed in only 9 of the 29 patients (31%), all after 2010. Four results were subnormal (<66%), and 2 were abnormal (<10%) (Table 1). Direct antiglobulin test was negative in all 10 patients reported by Shome et al. [3] and in all 7 of the other 19 patients with available results.

Twelve of 29 patients (41.4%) had dysfunction of ≥2 organs. Overall, 14 of 29 patients (48.3%) presented with acute chest syndrome [[3], [4], [5],^9^,^11^]. Sixteen of 29 patients (55.2%) required intensive care admission, and 13 (44.8%) needed ventilation. Renal dysfunction was observed in 11 of 29 patients (38%), with 1 requiring dialysis [15]. No sepsis was reported except 1 *Escherichia coli* bacteremia of unknown origin [21]. Chonat et al. [11] reported a case of TMA treated with plasma exchange (PLEX), but later found to have activation of the alternative complement pathway and genetic mutation that was treated with eculizumab.

The time from admission to TMA diagnosis in the 10 case series by Shome et al. [3], ranged from 1 to 20 days, with a median of 5 days. In the other 19 cases, it ranged from 2 to 5 days for 10 cases, on the first day in 6 cases, and after 6 days in 3 cases.

In the case series by Shome et al. [3], all 10 cases received red blood cell exchange (RCE) or packed red blood cell (PRBC) transfusions before TMA diagnosis [3]. Among the other 19 cases, 11 received packed PRBC transfusions, 8 underwent RCE, and 7 received both RCE and PLEX. Overall, 28 of 29 patients (97%) received PLEX. Two cases did not receive PLEX: 1 involved an old report of a patient with sickle cell trait treated with fresh frozen plasma, PRBC transfusion, and steroid [16], while the other was a case with Hgb SS treated with blood transfusion [19]. Intravenous immunoglobulin was given in 1 case [18], steroid in 5 patients, and rituximab in 1 patient [10]. Shome et al. [3] did not report the specific number of patients who received antibiotics. Among the other 19 patients, 9 (47.3%) received antimicrobial therapy. The average duration of PLEX was 7.5 days (SD ±3.7 days), with a median of 6.5 days for 8 patients. One patient underwent 5 sessions, and a second patient for 18 sessions; PLEX session numbers for 9 patients were unspecified. In the case series by Shome et al. [3], PLEX sessions ranged from 5 to 17, with a median of 10 [3].

In the 29 patients, there were 4 recurrences and 4 deaths. First death occurred 3 months after discharge due to another VOC without TMA [3]. The second death occurred from *Pneumocystis jirovecii* pneumonia 3 months after initial treatment with rituximab, steroid, and PLEX [10]; the third death occurred 2 months later following a recurrence [21]; and the fourth death occurred the next day after admission due to complications [18]. The overall fatality rate was 10.3%.

## 4 Discussion

TMA and aTTP (a subset of TMA with reduced ADAMTS13 activity) in this study appeared extremely rare, with only 29 reported cases over 36 years, perhaps from under recognition, under testing, and reporting bias. Inflammation, increased free Hgb from hemolysis, and hypoxia during a VOC can cause endothelial injury, leading to TMA and potentially aTTP through reduced ADAMTS13 activity. The diagnosis of TMA and aTTP is often delayed in these patients, underscoring the need for increased awareness and improved testing. Key laboratory indicators for both disorders include significantly elevated LDH, severe thrombocytopenia, declining Hgb, and the presence of schistocytes, with ADAMTS13 activity of <10% being specific to aTTP. Distinguishing between the 2 sometimes can be difficult, as aTTP may occur with a relative deficiency of ADAMTS13 (levels are >10%). Before diagnosing these disorders during a VOC, it is essential to rule out conditions such as splenic sequestration, aplastic crisis, or FES. We strongly recommend that all patients with suspected TMA or aTTP during a VOC undergo comprehensive testing, including assessment of ADAMTS13 activity and its inhibitors. Most patients had schistocytes on a smear, serving as a trigger for PLEX. However, identifying schistocytes could be challenging, as they may be absent or missed. Schistocyte may not appear until blood transfusion, delaying the diagnosis [6,22]. Treatment of patients in this study varied, PLEX being the most frequent approach, often combined with PRBC transfusions and RCE with good response. PLEX is clearly evidence-based effective therapy for patients with aTTP and ADAMTS13 level of <10%. In this review, patients with TMA including those with impaired ADAMTS13 activity (>10% to <66%) appeared to improve with PLEX. Caution may be required with steroid use in these patients, as it may be associated with adverse outcomes.

## 5 Conclusion

This review highlights that TMA including aTTP can arise in patients with SCD and its variants during a VOC, with symptoms overlapping with other differentials complicating the diagnosis. When a patient in VOC is strongly suspected to have TMA including aTTP, in the absence of clear alternative explanation, clinicians should initiate early PLEX irrespective of presence or absence of schistocytes. Similarly, when differentiating between FES and aTTP is challenging, early initiation of PLEX should be the first choice of treatment, as it may be effective in both conditions [23]. This study is constrained by the disease’s rarity, reliance on case reports, and potential diagnostic overlap with other conditions.
